# Predicting the influence of *Circ_0059706* expression on prognosis in patients with acute myeloid leukemia using classical statistics and machine learning

**DOI:** 10.3389/fgene.2022.961142

**Published:** 2022-10-21

**Authors:** Jichun Ma, Xiangmei Wen, Zijun Xu, Peihui Xia, Ye Jin, Jiang Lin, Jun Qian

**Affiliations:** ^1^ Deparrtment of Central Lab, Affiliated People’s Hospital of Jiangsu University, Zhenjiang, China; ^2^ Zhenjiang Clinical Research Center of Hematology, Affiliated People’s Hospital of Jiangsu University, Zhenjiang, China; ^3^ The Key Lab of Precision Diagnosis and Treatment in Hematologic Malignancies of Zhenjiang City, Affiliated People’s Hospital of Jiangsu University, Zhenjiang, China; ^4^ Deparrtment of Hematology, Affiliated People’s Hospital of Jiangsu University, Zhenjiang, China

**Keywords:** circ_0059706, acute myeloid leukemia, machine learning, prognosis, biomarker

## Abstract

**Background:** Various circular RNA (circRNA) molecules are abnormally expressed in acute myeloid leukemia (AML), and associated with disease occurrence and development, as well as patient prognosis. The roles of *circ_0059706*, a circRNA derived from *ID1*, in AML remain largely unclear.

**Results:** Here, we reported *circ_0059706* expression in *de novo* AML and its association with prognosis. We found that *circ_0059706* expression was significantly lower in AML patients than in controls (*p* < 0.001). Survival analysis of patients with AML divided into two groups according to high and low *circ_0059706* expression showed that overall survival (OS) of patients with high *circ_0059706* expression was significantly longer than that of those with low expression (*p* < 0.05). Further, female patients with AML and those aged >60 years old in the high *circ_0059706* expression group had longer OS than male patients and those younger than 60 years. Multiple regression analysis showed that *circ_0059706* was an independent factor-affecting prognosis of all patients with AML. To evaluate the prospects for application of *circ_0059706* in machine learning predictions, we developed seven types of algorithm. The gradient boosting (GB) model exhibited higher performance in prediction of 1-year prognosis and 3-year prognosis, with AUROC 0.796 and 0.847. We analyzed the importance of variables and found that *circ_0059706* expression level was the first important variables among all 26 factors included in the GB algorithm, suggesting the importance of *circ_0059706* in prediction model. Further, overexpression of *circ_0059706* inhibited cell growth and increased apoptosis of leukemia cells *in vitro*.

**Conclusion:** These results provide evidence that high expression of *circ_0059706* is propitious for patient prognosis and suggest *circ_0059706* as a potential new biomarker for diagnosis and prognosis evaluation in AML, with high predictive value and good prospects for application in machine learning algorithms.

## Introduction

Acute myeloid leukemia (AML) is one of the most common hematological malignancies and the most frequent type of acute leukemia in adults (Haubner et al., 2019; Newell and Cook, 2021). In recent years, treatment approaches, including molecular targeted therapy and hematopoietic stem cell transplantation, among others, have led to great progress in improving patient outcomes; however, 5-year survival rates remain low (Huang et al., 2019; Burchert et al., 2020; Vetrie et al., 2020; Du et al., 2021; Pollyea et al., 2021; Zhang et al., 2021). Therefore, research into the molecular mechanisms underlying the occurrence and development of AML is crucial to inform discovery of new clinical markers and therapeutic targets.

Circular RNA (circRNA) is a type of non-coding RNA characterized by a closed ring structure and circRNA molecules are widely distributed in eukaryotes, where they perform complex biological functions (Kristensen et al., 2019; Zhou et al., 2020). As circRNAs do not have 5′ end cap or 3′-polyadenylation tail structures, they cannot easily be recognized and degraded by RNase enzymes (Huang et al., 2020). Hence, circRNAs have high stability, as well as specificity, which contribute to its good potential for application in the field of tumor biomarkers (Xu et al., 2019; Wang et al., 2021; Wei et al., 2020). Some circRNA molecules are abnormally expressed in AML and associated with patient prognosis (Chen et al., 2018; Sun et al., 2019; Lin et al., 2021); for example, *circ-VIM* is significantly up-regulated in AML, and its over-expression is an independent prognostic factor associated with duration of overall and leukemia-free survival of patients with AML (Yi et al., 2019).

Developments in big data and computer hardware and software technologies have led to widespread use of machine learning in medicine (Ngiam and Khor, 2019; Radakovich et al., 2020). Compared with traditional statistics, machine learning has more powerful predictive ability (Lewis and Kemp, 2021; Tran et al., 2021) and its value for application in assisting disease diagnosis and predicting clinical outcomes has also attracted the attention of scholars.

Our previous study has revealed *ID1* transcript level significantly increased in AML and act as an independent risk factor in young non-M3 patients. *Circ_0059706* is a circular RNA, formed by *ID1* during its splicing. In this study, we investigated *circ_0059706* expression in patients with AML, evaluated its clinical significance, and analyzed the predictive ability of *circ_0059706* expression for AML prognosis using machine learning. The aim of the study was to explore the value of *circ_0059706* as a new tumor marker for predicting AML prognosis.

## Materials and methods

### Patients

This study was approved by the Ethics Committee of the Affiliated People’s Hospital of Jiangsu University and included 100 patients newly-diagnosed with AML and 33 healthy controls (K-20190020-Y). All samples were from the sample bank at our hospital and all patients signed informed consent forms. AML was classified according to World Health Organization (WHO) criteria and French-American-British (FAB) classification. Mutations were detected by high-resolution melting analysis (Wen et al., 2015).

### Cell culture and transfection

The K562 and THP-1 human leukemia cell line were purchased from ATCC. Cells were cultured in RPMI 1640 medium (Wisent, Nanjing, China) containing 10% fetal calf serum (FCS) (ExCell Bio, Shanghai, China) and 100 U/ml penicillin/streptomycin (Wisent, Nanjing, China) at 37°C in a 5% CO_2_ humidified atmosphere. Lentiviruses over-expressing *circ_0059706* were purchased from Shanghai Jikai Biological Co., Ltd. (Shanghai, China) and cell transfection performed according to the manufacturer’s instructions.

### Cell growth assays

Cells were seeded at 3 × 10^3^ per well in 96-well plates. After culture for 0, 24, 48, and 72 h, 10 μl CCK-8 (Dojindo, Kumamoto, Japan) solution was added to each well. Optical density values were measured at 450 nm absorbance using a microplate reader.

### Cell apoptosis assay

Cells (5 × 10^5^) were seeded into 6-well plates containing complete 1640 culture solution, without FCS, for 48 h. Apoptosis rate was determined using an apoptosis detection kit (Annexin V PE/7-AAD, BD Biosciences, Franklin Lakes, NJ, United States), and analyzed by flow cytometry on a FACSCalibur platform (Becton Dickinson, San Jose, CA, Unied States).

### RNA isolation, reverse transcription, and real-time quantitative PCR (RQ-PCR)

Mononuclear cells were extracted from bone marrow (BM) specimens using gradient centrifugation (TBD Sciences, China). RNA extraction and reverse transcription were conducted based on the instructions in miScript kits (Qiagen, Tilden, Germany). Reverse transcription and RQ-PCR were conducted as previously reported (Ma et al., 2014). The primers for *circ_0059706* were 5′-TGG​TAA​ACT​CTC​ATT​CCA​CGT​TC-3' (forward) and 5′-CCA​CTG​GCG​ACT​TTC​ATG​AT-3' (reverse). The primers used as controls were ABL and sequences were 5′- TCC​TCC​AGC​TGT​TAT​CTG​GAA​GA -3′ (forward) and 5′- TCCAACGAGCGGCTTCAC -3' (reverse). The primers for miR-326 were 5′-GTC​GTA​TCC​AGT​GCA​GGG​TCC​GAG​GTA​TTC​GCA​CTG​GAT​ACG​A CCTGGAG-3' (forward) and 5′- GCC​GAG​CCT​CTG​GGC​CCT​TC-3' (reverse). The primers for U6 were 5′-CTC​GCT​TCG​GCA​GCA​CA-3' (forward) and 5′-AAC​GCT​TCA​CGA​ATT​TGC​GT-3' (reverse). Relative *circ_0059706* expression levels were calculated using 2^−ΔΔCT^ method.

### Statistical analysis

Data were analyzed using SPSS 20.0 software. Relative levels of *circ_0059706* expression were calculated using the 2^−ΔΔCT^ method. Categorical variables were analyzed using chi square tests and/or Fisher’s exact tests. The diagnostic capacity of *circ_0059706* was analyzed using receiver operating characteristic (ROC) curves and area under the curve (AUC) values. Survival was analyzed using the Kaplan-Meier method. Univariate and multivariate Cox regression analyses were conducted. Differences in continuous variables between two groups were compared by Student’s t-test. Differences were considered statistically significant at two-tailed *p* < 0.05.

For machine learning, case and survival data, including 26 characteristic variables from 57 cases, were used. Twelve basic variables included in the analysis were: *circ_0059706* expression level, sex, age, white blood cell (WBC) count, hemoglobin (HB) level, platelet (PLT) count, BM blasts, diagnosis, karyotype chromosome abnormalities, chromosome risk group, blast percentage, and granulocyte count. Mutations of nine genes (*CEBPA*, *NPM1*, *FLT3*, *CKIT*, *RAS*, *IDH1/DH2*, *DNMT3A*, *SRSF2*, and *SETBP1*) were also included as variables. Five derivative variables and the methods used to generate them are shown in Table 1. Data from all cases were randomly divided into a training set (75%) for model development and a test set (25%) for performance evaluation. We developed seven types of machine learning algorithm, including: logistic regression (LR), random forest (RF), gradient boosting (GB), neural network (NNK), support vector machine (SVM), k-NearestNeighor (KNN), and Gaussian naïve Bayes (GNB). Parameters of 1-year survival and 3-year survival are as follows: C = 0.01 in LR, n_estimators = 300, random_state = 10 in RF, C = 100 in SVM, MLPClassifier (80, 10), random_state = 100 and random_state = 200 in NNK separately, n_estimators = 500, random_state = 300 and n_estimators = 200, random_state = 280 in GB separately, neighbors = 9 in KNN. Area under the ROC curve (AUROC), sensitivity, and specificity values were used as performance evaluation indicators. Machine learning algorithms were developed using Python software (version 3.7.6.). The “LogisticRegression,” “randomforestclassfier,” “gradientbootingclassifier,” “MLP classifier,” “SVC,” “Kneighborsclassifier,” and “Gaussian NB” functions in the “Sklearn” package (version 1.0.2.) were used for machine learning algorithms, and the “matplotlib” package was applied for machine learning data display.

**TABLE 1:** Methods used to generate the five derivative variables included in machine learning models.

Variable	Low group	High group
exp-group	Level of *circ_0059706* expression <0.254	Level of *circ_0059706* expression ≥0.254
age-group	Age <60 years	Age ≥60 years
WBC-group	WBC <30 × 10^9^/L	WBC ≥30 × 10^9^/L
Hb-group	HB < 110 g/L	HB ≥ 110 g/L
Plt-group	PLT <100 × 10^9^/L	PLT ≥100 × 10^9^/L

## Results

### *Circ_0059706* expression and its capacity to distinguish between patients with AML and healthy controls

**FIGURE 1:**
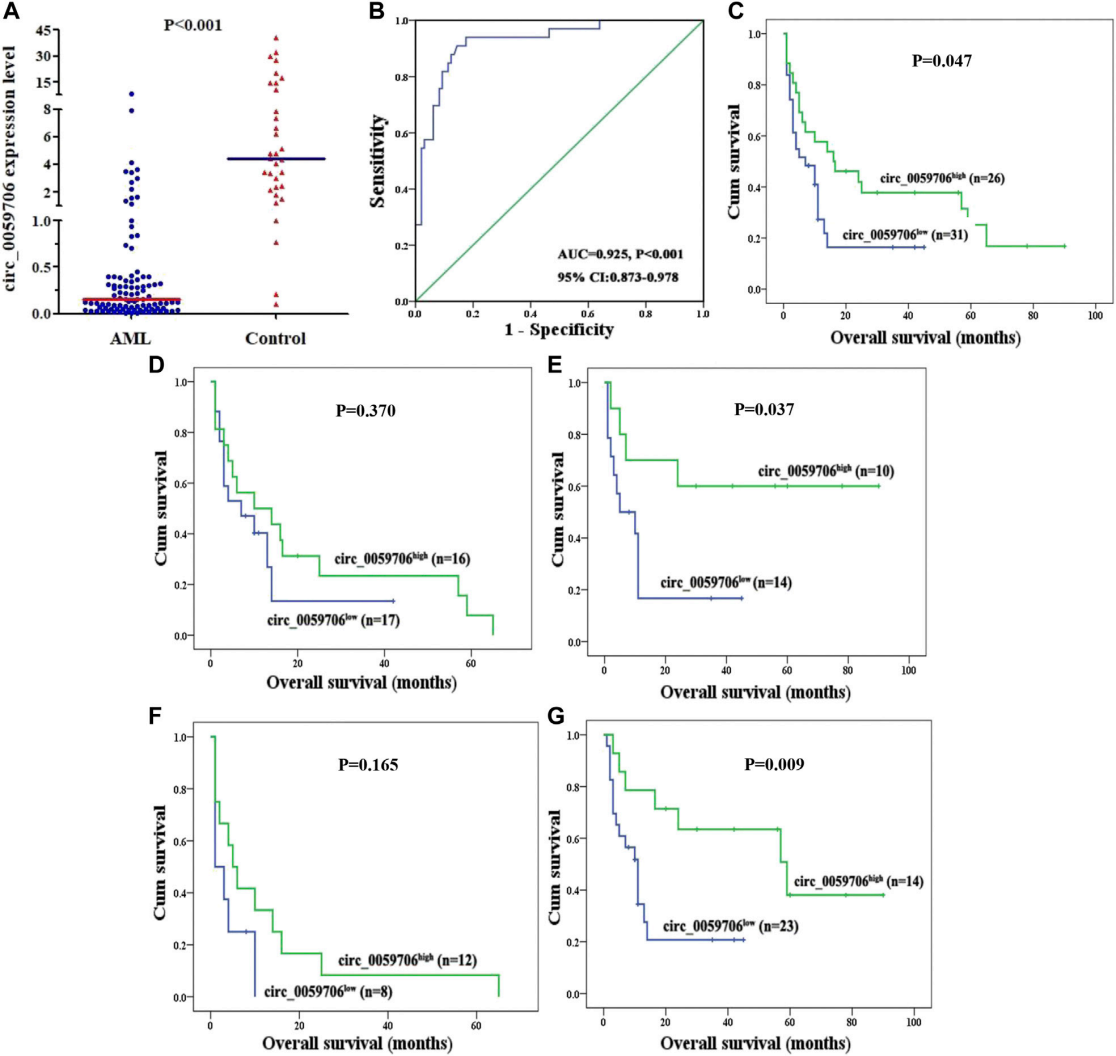
Expression of *circ_0059706* in patients with AML and its impact on overall survival. **(A)** Level of *circ_0059706* expression in controls and patients with AML detected by RQ-PCR. **(B)** Discriminative capacity of *circ_0057606* expression in patients with AML determined by ROC curve analysis. **(C–G)** Impact of *circ_0057606* expression on overall survival in: **(C)** total, **(D)** male, **(E)** female, **(F)** age >60 years, and **(G)** age ≤60 years, patients with AML.

Levels of *circ_0059706* expression in BM samples from 100 patients with AML and 33 healthy controls were detected by RQ-PCR. Median *circ_0059706* expression levels in healthy controls and patients with AML were 4.581 and 0.153, respectively; *Circ_0059706* expression was significantly lower in AML than in controls (*p* < 0.001) (Figure 1A). The AUC value of *circ_0059706* was 0.925 in AML patients (95% confidence interval: 0.873–0.978, *p* < 0.001) (Figure 1B), indicating that *circ_0059706* expression has potential as biomarker to distinguish AML from control samples.

### Associations between *Circ_0059706* expression and patient clinical characteristics

To investigate associations of *circ_0059706* expression with AML clinical characteristics, the total patient group was divided into *circ_0059706* ^high^ and *circ_0059706* ^low^ groups, according to median +1/16 standard deviation of *circ_0059706* expression level, using a cutoff value of 0.254 (sensitivity, 91.3%; specificity, 85.6%). Then, clinical parameters were compared between the high and low expression groups (Table 2). No significant differences were detected between the high and low expression groups; however, HB level, WBC count, and platelet count were higher in peripheral blood from the *circ_0059706* ^high^ group than from the *circ_0059706* ^low^ group, and the proportion of BM blast cells in the *circ_0059706* ^high^ group was lower than that of the *circ_0059706* ^low^ group. No correlations between gene mutations and *circ_0059706* expression were detected.

**TABLE 2:** Correlation between circ_0059706 expression and patients parameters.

Patient’s parameters	Status of circ_0059706 expression		
	Low (n = 57)	High (n = 40)	*p* value
Sex, male/female	29/28	25/15	0.354
Age, median (range), years^∆^	54 (18–84)	57 (10–81)	0.143
WBC, median (range), ×10^9^/L^∆^	37.7 (1.2–207.5)	59.3 (0.8–528.0)	0.124
Hemoglobin, median (range), g/L^∆^	84.9 (42–141)	93.2 (32–131)	0.099
Platelets, median (range),×10^9^/L^∆^	49.5 (4–192)	58.5 (4–382)	0.448
BM blasts, median (range), %^∆^	44.5 (0–94.5)	35.0 (0–95)	0.070
FAB classification			0.504
M0	1	0	
M1	2	0	
M2	23	18	
M3	8	5	
M4	13	9	
M5	4	7	
Risk classification			0.965
Low	13	9	
Intermediate	37	25	
High	7	4	
No data	0	2	
Karyotypes			0.661
normal	28	19	
*t(8;21)*	6	3	
*t(16;16)*	0	1	
*t(15;17)*	6	6	
*+8*	2	2	
*t(9;21)*	1	0	
*−7/7q-*	1	0	
Complex	6	3	
Others	7	4	
No data	0	2	
Gene mutations *			
*C-KIT (+/−)*	2/46	0/31	0.130
*FLT3 (+/−)*	4/44	5/26	0.147
*NPM1(+/−)*	12/37	5/28	0.156
*C/EBPA (+/−)*	5/43	1/31	0.116
*N/K-RAS (+/−)*	2/36	1/27	0.380
*IDH1/2 (+/−)*	0/48	1/31	0.115
*DNMT3A (+/−)*	2/46	2/30	0.343
*U2AF1 (+/−)*	0/48	0/32	
*CR(+/−)*	21/20	18/16	0.933

WBC, white blood cells; FAB, French-American-British classification; AML, acute myeloid leukaemia; CR, complete remission.

### Association of *Circ_0059706* expression level and prognosis of patients with AML

To explore association of *circ_0059706* expression with patient prognosis, we analyzed survival data from 57 patients. Although *circ_0059706* had no value for predicting complete remission (CR), patients in the *circ_0059706* ^high^ group had significantly longer overall survival (OS) than those in the *circ_0059706* ^low^ group (*p* = 0.047) (Figure 1C). Further, compared with male patients with AML, OS of *circ_0059706* ^high^ female patients was longer than that of *circ_0059706* ^low^ female patients (*p* = 0.037) (Figures 1D,E). Compared with older than 60 years, OS of those in the *circ_0059706* ^high^ group was significantly longer than that of those in the *circ_0059706* ^low^ group who were younger than 60-years-old AML (*p* = 0.009) (Figures 1F,G). Variables resulting in *p* < 0.1 in univariate analysis (age, WBC, risk classification, and *circ_0059706* expression) were included in multivariate analysis, which demonstrated that *circ_0059706* was an independent factor associated with poor prognosis in the total AML patients (*p* = 0.020) (Table 3).

**TABLE 3:** Univariate and multivariate analyses of prognostic factors for overall survival in whole-cohort AML patients.

Prognostic factors	Univariate		Multivariate	
	Hazard ratio (95% CI)	*p* value	Hazard ratio (95% CI)	*p* value
Age	2.557 (1.379–4.739)	0.003	2.519 (1.238–5.125)	0.011
Risk classification	2.265 (1.342–3.820)	0.002	1.527 (0.858–2.720)	0.150
circ_0059706 expression	0.529 (0.273–1.024)	0.059	0.340 (0.166–0.699)	0.003
WBC	2.482 (1.326–4.647)	0.004	2.077 (1.036–4.165)	0.040
Sex	0.628 (0.333–1.187)	0.152	---	---
*IDH1/2* mutations (+/−)	4.936 (0.625–38.974)	0.130	---	---
*NPM1* mutations (+/−)	1.767 (0.808–3.862)	0.154	---	---

+: positive; −: negative; *, +: bi-allelic mutation; −: mono-allelic mutation or wild type.

### Evaluation of prediction ability of *Circ_0059706* in machine learning algorithms

First, LR, RF, GB, NNK, SVM, KNN, and GNB 7 machine learning algorithms were developed using training set data, and their performance evaluated. As shown in Table 4, the GB model had better performance in predicting 1-year prognosis and 3-year prognosis, with AUROC 0.796 and 0.847, and sensitivity 0.615 and 0.923, specificity 0.75 and 1.

**TABLE 4:** Predictive performance comparison in the six kinds of machine learning algorithms.

	One year survival				Three years survival			
	AUROC	Sebsitivity	Specificity	Accuracy	AUROC	Sebsitivity	Specificity	Accuracy
LR	0.574	0.615	0.5	0.667	0.805	0.857	1	0.867
RF	0.722	0.6	0	0.6	1	0.8	0	0.8
GB	0.796	0.615	0.75	0.733	0.847	0.923	1	0.933
NNK	0.685	0.692	1	0.733	0.75	0.857	1	0.867
SVM	0.593	0.615	0.5	0.6	0.855	0.8	0	0.8
KNN	0.546	0.583	0.333	0.533	0.903	0.833	0	0.8
GNB	0.5	0.5	0.364	0.4	0.333	0.667	0	0.533

**FIGURE 2:**
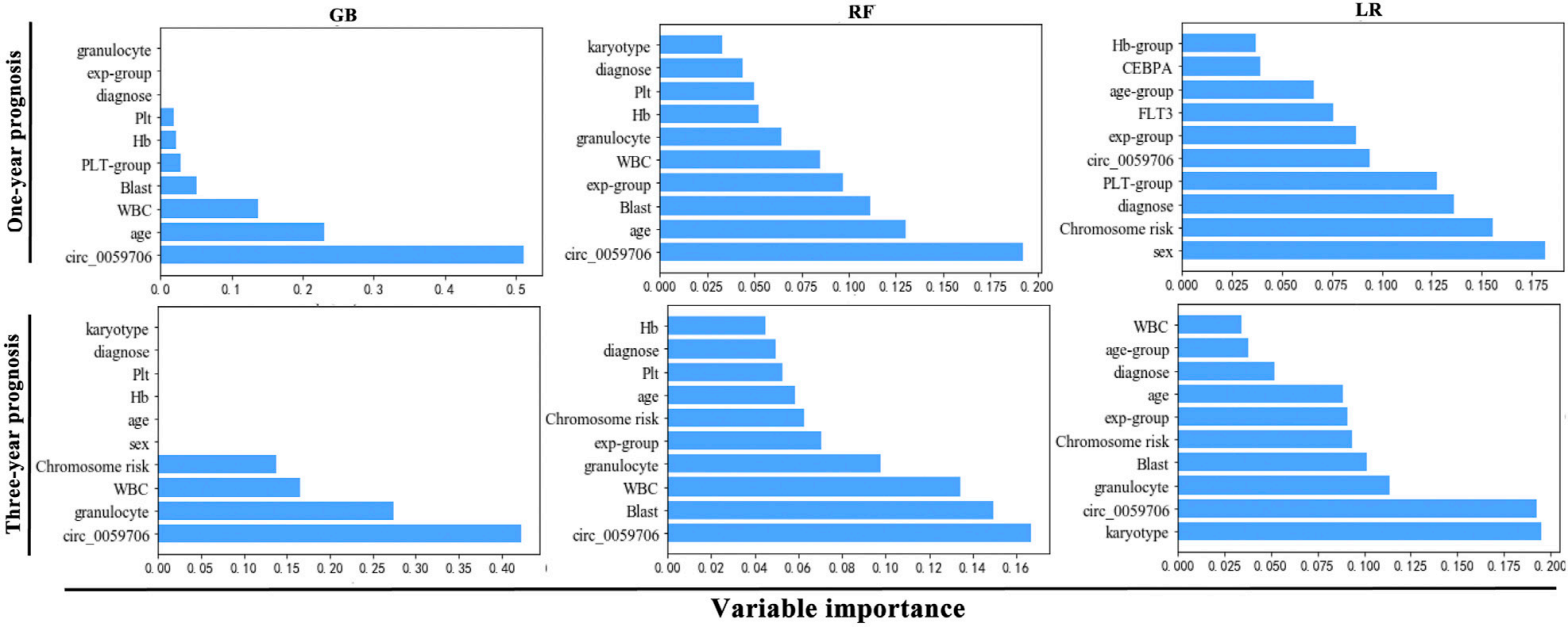
Top 10 variables most important in each algorithm.

The selection of variables for machine learning algorithms is critical. Therefore, we analyzed the importance of variables included in the GB, LR, and RF algorithms, which had good modeling performance. *Circ*_*0059706* expression level was the first important variable among all 26 features included in the GB and RF algorithms and was among the most important in the LR algorithm (Figure 2).

### *Circ_0059706* inhibits cell growth and increases apoptosis

**FIGURE 3:**
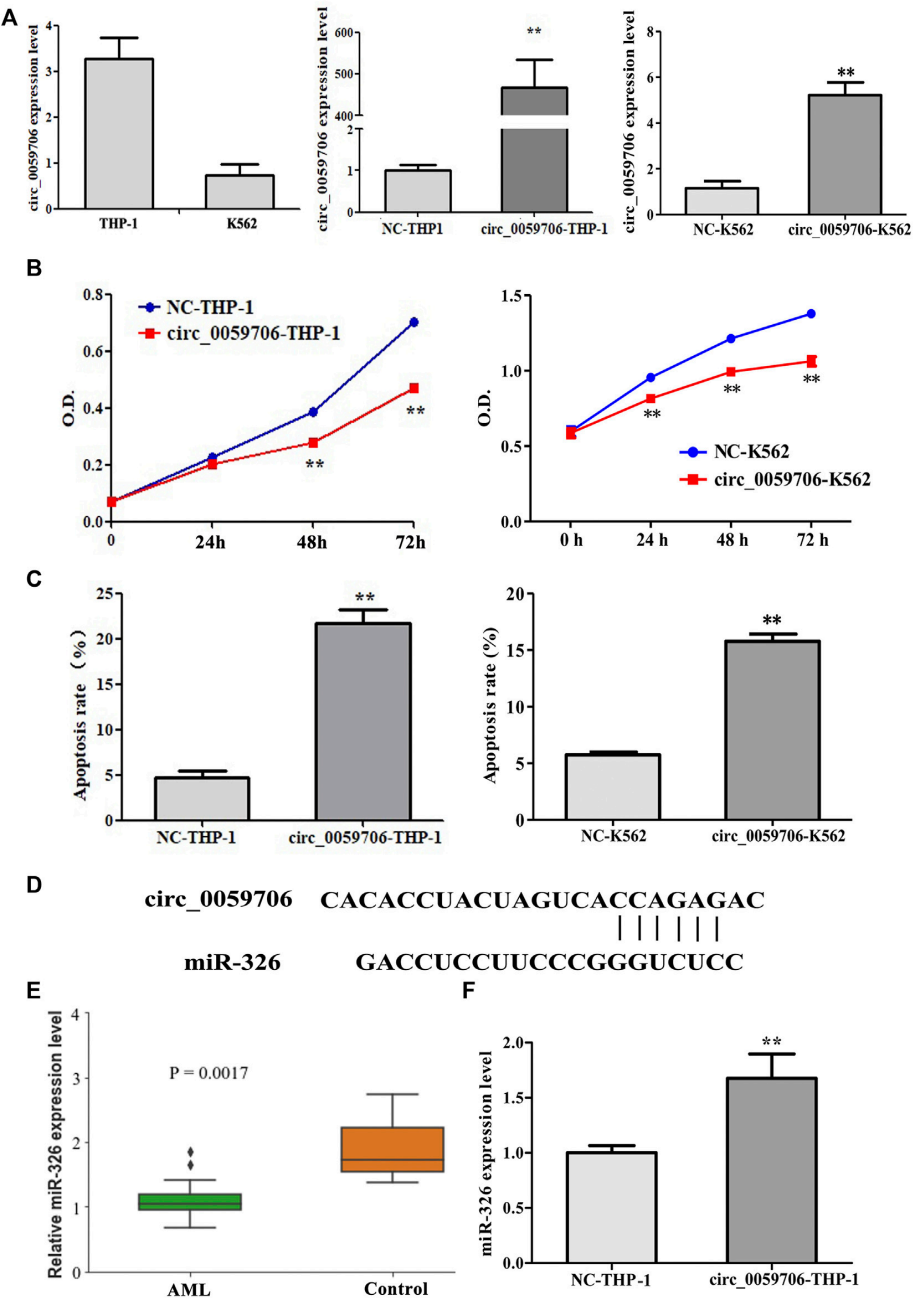
Effect of *circ_0059706* on cell growth and apoptosis. **(A)** The level of *circ_0059706* expression was detected by RQ-PCR. **(B)** *Circ_0059706* inhibited cell growth in THP-1 and K562 cells detected by CCK8. **(C)** *Circ_0059706* promoted cellular apoptosis in THP-1 and K562 cells, as detected by flow cytometry. **(D)** Bioinformatics analysis of the binding site between circ_0059706 and miR-326. **(E)** Expression levels of miR-326 in controls and AML patients were analyzed in the GSE51908 datasets. **(F)** The level of miR-326 was detected in *circ_0059706* overexpressed cells by RQ-PCR. **, compared with the NC group, *p* < 0.01.

To study the effect of *circ_0059706* on leukemia cells, we over-expressed it in THP-1 and K562 cells (Figure 3A), and found that cell growth rate was inhibited by *circ_0059706* over-expression (Figure 3B). Moreover, the apoptosis rate of *circ_0059706*-transfected THP-1 and K562 cells was significantly higher than that of the control group (*p* < 0.01) (Figure 3C).

To determine the possible mechanism involved in the functionality of *circ_0059706*, we performed additional analysis. We analyzed the miRNAs that may bind to *circ_0059706* by use of the circinteratome database (https://circinteractome.nia.nih.gov/index.html). Then, the expression levels of these miRNAs in AML patients were analyzed by the GEO database (Datasets: GSE51908). Bioinformatics analysis revealed that *circ_0059706* contains a binding site for miR-326 (Figure 3D). The levels of *miR-326* were lower in AML patients when compared with controls in GSE51908 datasets (Figure 3E). Finally, the expression levels of *miR-326* were found to be up-regulated in cells that overexpressed *circ_0059706* (Figure 3F).

## Discussion

CircRNAs are non-coding RNAs that have recently emerged as a potential tumor biomarkers and drug targets, with good prospects for clinical application. *CircPLXNB2* is a valuable predictor of prognosis in patients with AML (Lin, Wang, Bian, Sun, Guo, Kong, Zhao, Guo, Li, Wu, Wang, Wang and Li, 2021). Further, Liu et al. (2022) found that expression of *hsa_circ_0004277* can be restored after chemotherapy in patients with AML, suggesting that its up-regulation is associated with successful treatment; hence, *hsa_circ_0004277* is a potential diagnostic marker and treatment target in AML. Further, *circRNF220* can distinguish AML from ALL and other hematological malignancies, while high *circRNF220* expression is an unfavorable prognostic marker of recurrence, due to its role in sequestration of miR-30a, which increases MYSM1 and IER2 expression and is implicated in AML relapse (Liu et al., 2021).

*Circ_0059706* derived from *ID1*. *ID1* is a negative regulator of the HLH transcription factor that plays the role of an oncogene in promoting cell cycle, proliferation and inhibiting apoptosis (Yin et al., 2017; Chen et al., 2020). Our group previously reported that *ID1* expression was up-regulated in AML patients, and the high expression associated with poor prognoses. Here, we found that *circ_0059706* was down-regulated in AML. HB, WBC, and PLT levels were higher in peripheral blood from the *circ_0059706* ^high^ group than those in the group with low *circ_0059706* expression. Further, the proportion of BM blast cells in the *circ_0059706* ^high^ group was lower than that in the *circ_0059706* ^low^ group. Hence, routine blood parameters appeared to be better in the high expression group than those of the low expression group. There were no correlations between gene mutations and *circ_0059706* expression. Survival analysis showed that *circ_0059706* has no value for prediction of CR; however, OS was significantly higher in patients with high *circ_0059706* expression than those with low expression. Longer OS was observed in female patients and those >60-years-old with high *circ_0059706* expression than in male patients <60-years-old. Furthermore, the total patient group was divided into *circ_0059706* ^high^ and *circ_0059706* ^low^ groups, according to circ_0059706 expression level at quartiles or tertiles. Patients in the *circ_0059706* ^high^ group had significantly longer OS than those in the *circ_0059706* ^low^ group (*p* = 0.038, *p* = 0.027). Multivariate analysis showed that *circ_0059706* low expression was an independent factor associated with poor prognosis of all patients with AML, indicating that *circ_0059706* has potential for application as a new biomarker for diagnosis and prognosis evaluation of AML.

Traditional statistics are generally used to infer relationships between variables, while machine learning models aim to make the most accurate predictions possible, and are increasingly being applied in medical prediction models. Gao et al. predicted a significant association between Luminal and HER2 breast cancer subtypes and estrogen/progesterone and HER2 receptor status, using the DeepCC method (Gao et al., 2019). Lee et al. comprehensively analyzed RNA-seq data and identified a potential role for machine learning in identifying categories of acute leukemia (Lee et al., 2021). Based on traditional statistical analysis, we found that *Circ*_*0059706* level are closely related with survival, suggesting its potential as a biomarker in patients with AML. Different machine learning algorithms may be optimal for any data set; therefore, to assess the prospects for application of *circ*_*0059706* levels in AML in machine learning algorithms, we developed seven types of machine learning algorithm, including LR, RF, GB, NNK, SVM, KNN, and GNB. The GB model had better performance in predicting 1-year prognosis and 3-year prognosis. We were unable to predict 5-year survival due to insufficient data. In recent years, scholars have established many risk assessment methods in various disease prognosis models using machine learning, which can provide guidance for the selection of treatment methods and prognosis assessment. For example, Heo applied an NNK algorithm to establish a prediction model for long-term prognosis in patients following ischemic stroke (Heo et al., 2019), while Tian established an early gastric cancer lymph node metastasis prediction model using a regularized dual averaging approach (Tian et al., 2021). Moreover, machine learning achieved acceptable prediction of central lymph node metastasis, with a GB model performing best, which may help to determine the optimal extent of initial surgical treatment for patients with T1–T2 stage, non-invasive, and clinically node-negative papillary thyroid cancer (Zhu et al., 2021).

Variables are crucial to the prediction results generated by machine learning; hence, the key roles of variables included in the machine learning models was also a focus of our attention. We analyzed the importance of variables in the GB, LR, and RF algorithms, which had good modeling performance. *Circ_0059706* expression level was the first important variable among all 26 features included in the GB and RF algorithms, and it ranked highly in the LR algorithm. It indicated that *circ_0059706* has a high predictive value and a good prospect for application in machine learning, supporting the potential of this circRNA as a new biomarker for diagnosis and prognosis evaluation in AML.

Furthermore, we analyzed the effect of *circ_0059706* on cell growth and apoptosis in leukemia cells. The results showed that *circ_0059706* overexpression inhibited cell growth and increased apoptosis, further supporting the hypothesis that the high expression of this circRNA is propitious for patient prognosis. To investigate the mechanisms, we analyzed miRNAs with common binding sites for *circ_0059706* in the circinteratome database. The expression levels of miRNAs were analyzed by datasets GSE51908. Combined with literature reports, we focused *miR-326*, which was downregulated in GSE51908 datasets. P Cheng reported that expression of *miR-326* was downregulated in AML patients compared with that in normal. Overexpression of *miR-326* inhibited proliferation, promoted cell apoptosis and PMA-induced differentiation in AML cells (Cheng et al., 2020). Moreover, *miR-326* down regulated in ALL patients and negative associated with its expression and MDR (Ghodousi and Rahgozar, 2018). These results suggested *miR-326* maybe act as a tumor suppressor miRNA in leukemia and it was upregulated in *circ_0059706* over-expressed cells. *miR-326* was up-regulated in *circ_0059706* overexpression cells, it may be a mechanism of inhibited growth and promoted cell apoptosis. However, more experiments needed to verify, such as luciferase reporter experiment, the effect of up/down-regulation of *miR-326* expression on cell biological function, etc.

## Conclusion

Taken together, our results indicate that down-regulation of *circ_0059706* is a frequent event and predicts poor prognosis in patients with *de novo* AML. *Circ_0059706* showed good predictive effects in machine learning models and was among the most important variables in the developed models. In addition, *circ_0059706* overexpression could inhibit cell growth and increase apoptosis. These results demonstrated that *circ_0059706* might act as a potential biomarker for prognosis in *de novo* AML.

## Data Availability

The original contributions presented in the study are included in the article/supplementary material, further inquiries can be directed to the corresponding authors.
